# Study protocol: the JEU cohort study – transversal multiaxial evaluation and 5-year follow-up of a cohort of French gamblers

**DOI:** 10.1186/s12888-014-0226-7

**Published:** 2014-08-20

**Authors:** Gaëlle Challet-Bouju, Jean-Benoit Hardouin, Jean-Luc Vénisse, Lucia Romo, Marc Valleur, David Magalon, Mélina Fatséas, Isabelle Chéreau-Boudet, Mohamed-Ali Gorsane, JEU Group, Marie Grall-Bronnec

**Affiliations:** Clinical Investigation Unit BALANCED “BehaviorAL AddictioNs and ComplEx mood Disorders”, Department of Addictology and Psychiatry, University Hospital of Nantes, 85 rue de Saint Jacques, 44093 Nantes Cedex 1, France; EA 4275 SPHERE “bioStatistics, Pharmacoepidemiology and Human sciEnces Research tEam”, Faculties of Medicine and Pharmaceutical Sciences, University of Nantes, Paris, France; Unit of Methodology and Biostatistics, University Hospital of Nantes, Paris, France; EA 4430 CLIPSYD « CLInique PSYchanalyse Développement », University of Paris Ouest Nanterre La Défense, Paris, France; Louis Mourier Hospital of Colombes, Assistance Publique – Hôpitaux de Paris (APHP), Paris, France; Present address: Psychotherapies Unit, Sainte-Anne Hospital – Psychiatry and Neurosciences, Paris, France; Marmottan Medical Center, GPS Perray-Vaucluse, Paris, France; Department of Adult Psychiatry, Sainte-Marguerite University Hospital of Marseille, Paris, France; Psychiatry Laboratory, Sanpsy CNRS USR 3413, University of Bordeaux and Charles Perrens Hospital, Bordeaux, France; Psychiatry Department, University Hospital of Clermont-Ferrand, Paris, France; Psychiatry and Addictology Department, Paul Brousse University Hospital of Villejuif, Assistance Publique – Hôpitaux de Paris (APHP), Paris, France; Present address: Addictology Department, University Hospital Group Henri Mondor of Creteil, Paris, France

**Keywords:** Gambling, State changes, Cohort, Problem gambling, Recourse to treatment, Predictive factors

## Abstract

**Background:**

There is abundant literature on how to distinguish problem gambling (PG) from social gambling, but there are very few studies of the long-term evolution of gambling practice. As a consequence, the correlates of key state changes in the gambling trajectory are still unknown. The objective of the JEU cohort study is to identify the determinants of key state changes in the gambling practice, such as the emergence of a gambling problem, natural recovery from a gambling problem, resolution of a gambling problem with intermediate care intervention, relapses or care recourse.

**Methods/design:**

The present study was designed to overcome the limitations of previous cohort study on PG. Indeed, this longitudinal case–control cohort is the first which plans to recruit enough participants from different initial gambling severity levels to observe these rare changes. In particular, we plan to recruit three groups of gamblers: non-problem gamblers, problem gamblers without treatment and problem gamblers seeking treatment.

Recruitment takes place in various gambling places, through the press and in care centers.

Cohort participants are gamblers of both sexes who reported gambling on at least one occasion in the previous year and who were aged between 18 and 65. They were assessed through a structured clinical interview and self-assessment questionnaires at baseline and then once a year for five years. Data collection comprises sociodemographic characteristics, gambling habits (including gambling trajectory), the PG section of the DSM-IV, the South Oaks Gambling Screen, the Gambling Attitudes and Beliefs Survey – 23, the Mini International Neuropsychiatric Interview, the Wender-Utah Rating Scale-Child, the Adult ADHD Self-report Scale, somatic comorbidities (especially current treatment and Parkinson disease) and the Temperament and Character Inventory – 125.

**Discussion:**

The JEU cohort study is the first study which proposes to identify the predictive factors of key state changes in gambling practice. This is the first case–control cohort on gambling which mixes non-problem gamblers, problem gamblers without treatment and problem gamblers seeking treatment in almost equal proportions. This work may help providing a fresh perspective on the etiology of pathological gambling, which may provide support for future research, care and preventive actions.

**Trial Registration:**

(ClinicalTrials.gov): NCT01207674.

**Electronic supplementary material:**

The online version of this article (doi:10.1186/s12888-014-0226-7) contains supplementary material, which is available to authorized users.

## Background

Pathological Gambling (PG) is a behavioral addiction characterized by a loss of control over gambling which then becomes the subject’s only interest, prevailing over all his/her other activities, causing serious harmful consequences to social, family, or financial life. The prevalence of lifetime PG is estimated at around 0.4-1.0% [1].

There is abundant literature on how to distinguish problem gambling from social gambling [2-6], but there are very few studies of the long-term evolution of gambling practice, even though this study design is the only one which can identify protective and risk factors for PG [7]. We can mention the study by Slutske in 2003 [8], from which the main lesson is that gambling problems are often transient and episodic rather than continuous and chronic. Other studies confirmed that a status of pathological gambler is unstable over time (16–19). Another cohort study by Nelson et al. [9] showed that the onset of gambling and gambling problems occurs later in women, but they seek treatment sooner. Another important result is that the earlier initiation occurs, the longer the time between initiation and recourse to treatment, for both genders. Although these studies are particularly interesting for understanding the gambling trajectory, they have several limitations. The main one is the limited samples used (students [8], pathological gamblers in treatment [9], males from 45 to 60 years old [10], young adults [11], casino employees [12], etc.). Those populations are not representative of the gamblers’ population as a whole. There are very few samples that combine both non-problem gamblers, problem gamblers who are not undergoing treatment and problem gamblers seeking treatment, in sufficient proportions for analysis of the gambling trajectory. Moreover, these studies did not try to identify the protection or risk factors which determine changes of state in the gambling trajectory. The majority of studies only report the description and prevalence of these state changes, but do not look for a causal effect between certain psychosocial correlates and changes over time. When these studies explored these state changes in greater detail, they were limited by the restricted number of problem gamblers in their samples, making it difficult to observe the changes [13]. Moreover, most of the studies that attempted to explore the natural history of gambling used retrospective data, collected cross-sectionally [9,10,13]. Longitudinal studies are still rare, and are subject to the other above-mentioned limitations [12] or do not explore all the possible state changes [14]. Finally, most of these studies are based exclusively on self-reported measurements of gambling practice and PG symptoms [9-11].

Therefore, the correlates of key state changes in the gambling trajectory are still unknown. The overall objective of the JEU cohort study is to understand how and why a gambling practice evolves. The study aims:

1. to explore and describe the gambling population, especially specific profiles based on: their sociodemographics (especially gender), their gambling habits (gambling trajectory, gambling activities, gambling-related cognitions, etc.) and their psychiatric comorbidities (including Attention Deficit Hyperactivity Disorder (ADHD)).
2. to compare gamblers at baseline, depending on whether they are problem or non-problem gamblers, and included or not in a treatment program. The aim of doing this is to isolate factors which may differ depending on the presence of a gambling problem and the recourse to treatment, to test them as potential determinants of the evolution of the gambling practice.
3. to identify longitudinal predictors of five key state changes: emergence of a gambling problem, natural recovery from a gambling problem, resolution of a gambling problem with intermediate care intervention, relapses and recourse to treatment.

## Methods/design

### Setting of the study – consortium

This cohort was established in 2009 and is coordinated by two researchers (first and last author) from the Clinical Investigation Unit BALANCED “*BehaviorAL AddictioNs and ComplEx mood Disorders*” at the University Hospital of Nantes and the SPHERE research team “*bioStatistics, Pharmacoepidemiology and Human sciEnces Research tEam*” at the University of Nantes. The University Hospital of Nantes is the sponsor of this study. The study involves a group of French clinicians and researchers from seven French institutions which have a care offer or a research area dedicated to PG (Northwestern: University Hospital of Nantes associated with University of Nantes; Southwestern: University of Bordeaux associated with Charles Perrens Hospital of Bordeaux; Paris region: University of Paris Ouest Nanterre La Défense associated with Louis Mourier Hospital of Colombes, Marmottan Medical Center in Paris, Paul Brousse University Hospital of Villejuif; Southeastern: Sainte-Marguerite University Hospital of Marseille; Center: University Hospital of Clermont-Ferrand).

### Study design

The present study was designed to overcome the limitations of previous cohort study on PG. In particular, it was very important to have enough participants in the initial groups to observe these rare changes. We thus designed a longitudinal case–control cohort, which was divided in two phases:

Phase 1 aims to constitute a large sample of gamblers and compare three groups: Non-Problem Gamblers (NPG), Problem Gamblers Without Treatment (PGWT) and Problem Gamblers Seeking Treatment (PGST). This phase consists of a baseline assessment.

**Figure 1:**
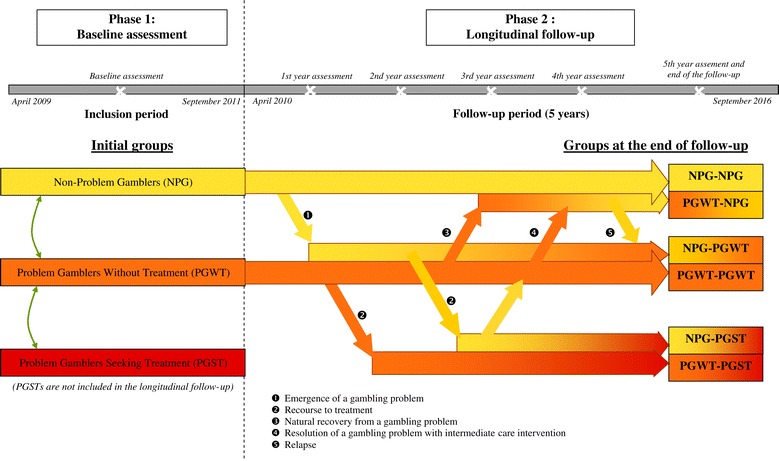
**Study design.**

Phase 2 is the key step of the study which aims to study the differential long course development of NPGs and PGWTs. This longitudinal part of the study consists of five years prospective follow-up. Since the future of problem gamblers in care was not one of our objectives, PGSTs were not included in the longitudinal follow-up. By following the evolution of socio-demographic and clinical variables in addition to these state changes, we will be able to identify the predictive factors of these state changes. An illustration of the study design is given in Figure 1.

### Ethical approval

Participants were informed about the research and gave their written informed consent prior to their inclusion in the study. This study was approved by the French Research Ethics Committee (CPP) on January 8, 2009. The approval granted from the CPP applies to all sites where the study takes place.

### Participants

Gamblers of both sexes who reported gambling on at least one occasion during the previous year, and aged between 18 and 65 were eligible for the study. Exclusion criteria were severe cognitive or communication impairment and guardianship. Recruitment took place between April 2009 and September 2011, in five regions of France (Northwest, Southwest, Paris region, Center and Southeast). NPGs and PGWTs were recruited in various gambling places (casinos, cafés, smoke shops, etc.) and through the press, in order to obtain the broadest possible range of gambling severity levels and gambling activities. PGSTs were recruited in seven care centers. Participation in the study was proposed during the inclusion period to each new patient and to patients who had started treatment less than six months before. Of the 628 eligible volunteers who agreed to take part in the study, 206 were recruited in care centers, 195 in gambling places and 227 through the press.

### Sample size

The computation of the number of subjects to be included was based on the assumption that the presence of psychiatric comorbidities was a determining factor in the evolution of gambling practice. Thus, for phase 1, the inclusion of between 500 and 680 subjects was intended to highlight a minimum difference of three psychiatric comorbidities between groups, with a power of at least 90% and a bilateral risk α of 5%. For phase 2, information from the scientific literature and cohort studies conducted previously were too fragmentary at the time of study design to allow us to estimate correctly the probability of state changes. As a consequence, a formal computation of the number of subjects required in follow-up wasn’t performed. Because of the low prevalence of gambling problems in the general population, the sample was constituted based on a predetermined and approximate equality of size between NPGs, PGWTs and PGSTs (that is between 160 and 260 participants per group).

### Procedure

A baseline assessment is performed just after inclusion in the study. The proposed assessment mixes a clinical structured interview carried out with a trained researcher or psychologist with a set of standardized self-report questionnaires. Participants realize the baseline interview in the research center or the gambling place in which they were recruited. Participants from the initial NPG and PGWT groups are followed up for 5 years after their inclusion. Participants are contacted once each year on the anniversary date of the last completed assessment (plus or minus 2 months). The follow-up interview is offered by phone or at the research center, depending on the availability and desire of the participant. The objective is to propose simple follow-up modalities to maintain the highest number of participants in the study. If a follow-up assessment does not take place, the reason is postponed (unreachable, refusal to continue, or death). For unreachability, a participant is considered to have withdrawn from the study only if two consecutive assessments are missing. Each assessment (baseline and follow-up assessments) comprises almost the same questionnaire content, which is described below.

### Measures

#### Inclusion of variables to be monitored over time

The final choice of clinical and gambling-related variables to be monitored over time was based on discussions with the teams participating in the project (JEU group) and a review of the literature. We chose to restrict the assessment procedure to a limited number of questionnaires, in an attempt to reduce the duration of the assessment to a maximum of one thirty. We expected that the shorter the duration of the assessment, the more the procedure would be accepted by the gamblers, especially PGWTs who are particularly difficult to recruit for research purposes.

The proposed assessment content was tested in a preliminary feasibility study, in particular within the population of gamblers recruited in gambling places. This study allowed us to estimate the human resources needed to achieve effective recruitment set (inclusion period of a year per center), and favor interview modalities with the lowest rate of missing data (interview in the research center).

The final set of questionnaires includes socio-demographic characteristics, gambling-related data, psychiatric and somatic comorbidities, ADHD antecedents and personality profile. The complete assessment content is detailed in Table 1.

**Table 1:** **Content of the multiaxial assessment for each phase of follow-up**

**Phase**	**Measurements**
**Baseline** NPG, PGWT and PGST	**Informed written consent**
	**Clinical structured interview (lifetime and past year):**
	-Sociodemographic characteristics
	-PG section of DSM-IV (diagnosis of a gambling problem)
	-Gambling habits
	-MINI (psychiatric and addictive comorbidities)
	-Somatic comorbidities
	**Self-report questionnaires:**
	-SOGS (self-reported severity of gambling problems)
	-GABS-23 (gambling-related cognitions)
	-TCI-125 (temperament and character dimensions)
	-WURS-C (screening of ADHD in childhood)
	-ASRS-1.1 (screening of ADHD in adulthood)
**Follow-up each year** Initial NPG and PGWT	**Clinical structured interview (past year):**
	-Sociodemographic characteristics
	-PG section of DSM-IV (diagnosis of a gambling problem)
	-Gambling habits (without gambling course)
	-MINI (psychiatric and addictive comorbidities)
	-Somatic comorbidities
	**Self-report questionnaires:**
	-SOGS (self-reported severity of gambling problems)
	-GABS-23 (gambling-related cognitions)
	-TCI-125 (only character dimensions, i.e. 65 items)
**Ongoing**	All participants are monitored using routine data sources providing deaths and study dropouts (unreachable or refusal to continue) since baseline**.**

*Notes:*

*NPG* = non-problem gamblers, *PGWT* = problem gamblers without treatment, *PGST* = problem gamblers seeking treatment.

*PG* = pathological gambling.

*ADHD* = Attention Deficit Hyperactivity Disorder.

*DSM-IV* = Diagnostic and Statistical Manual of Mental Disorders.

*MINI* = Mini International Neuropsychiatric Interview – fifth version.

*SOGS* = South Oaks Gambling Screen.

*GABS-23* = Gambling Attitudes and Beliefs Survey - Revised version.

*TCI-125* = Temperament and Character Inventory – 125.

*WURS-C* = Wender-Utah Rating Scale-Child.

*ASRS-1.1* = Adult ADHD Self-report Scale.

#### Categorization between problem and non-problem gamblers

The distinction between problem and non-problem gamblers was achieved through an interview based on the DSM-IV [15] 10 diagnostic criteria for PG. Gamblers who met at least three DSM-IV criteria were classified as problem gamblers (including both gamblers “at risk” for pathological gambling and gamblers with a diagnosis of PG), and those remaining as non-problem gamblers. We used a non-standard threshold of three instead of five to include subclinical forms of PG. Previous literature has supported the relevance of this categorization [16-18]. The number of positive DSM IV criteria for pathological gambling is also used as a dimensional score of gambling problem severity and the responses to each criterion is taken into account to study the various symptoms of pathological gambling.

#### Gambling-related characteristics

Participants were asked about participation in various forms of gambling over the past year, monthly gambling expenditure especially in relation to income, maximum wagering in a single day, age at which they were initiated into gambling and family history of problem gambling. They were also invited to determine their preferred gambling activity, i.e. the one which they preferred among all the gambling activities they have experimented in their lives (gamblers with a multi-game profile were restricted to defining a single preferred gambling activity). They also completed two self-report questionnaires related to gambling. The South Oaks Gambling Screen (SOGS) [19] is used to assess the severity of gambling problems. The Gambling Attitudes and Beliefs Survey (GABS) [20] is used to assess irrational beliefs and attitudes about gambling. The GABS-23 [21] is a revised version of the original GABS which consists of 23 items divided into 5 dimensions: Strategies, Chasing, Attitudes, Luck and Emotions.

#### Psychiatric and somatic comorbidities

The main axis-I psychiatric disorders were explored with the Mini International Neuropsychiatric Interview (MINI 5.0) [22]. It includes assessment of the major anxiety disorders, mood disorders (plus current risk of suicide), addictive disorders and to a lesser extent psychotic disorders. Somatic comorbidities were explored by asking the participants about their current medications and possible somatic pathologies (especially dopaminergic medications for Parkinson disease [23-25] and aripiprazole for schizophrenia or bipolar disorder [26,27]).

#### ADHD antecedents

Two self-report questionnaires are used to screen ADHD in the sample. The Wender-Utah Rating Scale-Child (WURS-C) [28,29] is used to make a retrospective screening of ADHD in childhood, and is supplemented by the Adult ADHD Self-report Scale (ASRS-v1.1) [30,31] which screens ADHD in adulthood.

#### Personality profile

The shorter 125-item version of the Temperament and Character Inventory (TCI-125) [32,33] is used to rapidly explore the seven dimensions of personality defined by Cloninger’s psychobiological model [32]. The TCI-125 assesses four temperament traits (Novelty Seeking, Harm Avoidance, Reward Dependence and Persistence) and three character traits (Self-Directedness, Cooperation and Self-Transcendence). Moreover, the optional section for antisocial personality disorder of the MINI [22] is also used to make a diagnosis of antisocial personality disorder. It will not be taken into account for the calculation of the number of psychiatric comorbidities (which is one of our major hypothetic predictor of the gambling practice evolution), since it is not an axis I psychiatric comorbidity but an element of personality (Axis II).

#### Outcome measures

The primary outcome measures for the JEU cohort are change in gambling status (problem or non-problem gambling) and recourse to treatment. The presence of a gambling problem is assessed annually with the 10 criteria from the DSM-IV (see above). Recourse to treatment is also assessed annually through one question: “Did you consult a health professional for a gambling problem in the past twelve months?”. A change in one of these two measures during the follow-up is considered as a state change in the gambling history.

### Analysis

Descriptive analysis of all the variables collected at baseline will be provided, for the whole sample and per group. A summary of the major characteristics of the sample at baseline is shown in Table 2. Description of loss of follow-up (drop-out, missing data) will also be provided, especially reasons for drop-out.

**Table 2:** **Major characteristics of the JEU Cohort (n = 628) at baseline**

	**N (responders)**	**%**	**M (SD)**
**SOCIODEMOGRAPHICS**			
Gender (males)	628	66.6%	
Age (years)	627		43.4 (12.9)
Monthly income (€)	623		1739.1 (1957.4)
Professional activity (working)	627	63.5%	
Marital status (living alone)	627	49.9%	
**GAMBLING-RELATED CHARACTERISTICS**			
**Gambling status**	628		
Non-Problem Gamblers (NPG)		40.8%	
Problem Gamblers without Treatment (PGWT)		26.9%	
Problem Gamblers Seeking Treatment (PGST)		32.3%	
**Preferred gambling activity**	628		
Electronic gaming machines (EGM) (*Slots, videopoker*)		26.1%	
Horserace betting		21.3%	
Poker		12.4%	
Scratch cards		12.4%	
Lottery games with differed results (*Loto®* ^*1*^ *, Euromillions®* ^*2*^ *, Kéno ®* ^*1*^)		11.1%	
Sports betting		7.6%	
Roulette		3.8%	
Lottery games with instant results (*Rapido®* ^*3*^ *, online Bingo*)		2.5%	
Black Jack		0.5%	
Non classified (name given too sketchy)		2.1%	
Frequency of gambling (*once a week or more*)	628	76.0%	
Monthly gambling expenditure (€)	621		592.7 (1494.4)
Proportion of income spent on gambling	612	35.3%	
Maximum amount wagered in one day (€)	605		1275.2 (5349.0)
Age of initiation into gambling	628		20.4 (9.3)
Age of onset of a regular gambling practice	584		26.4 (11.4)
Age of onset of gambling problems (PGWT and PGST only)	350		34.7 (11.7)
Age of first consultation for gambling problems (PGST only)	196		40.1 (11.3)
Internet as the favorite medium of gambling	571	12.6%	
Experience of abstinence for one month or more	627	62.7%	
Family aware of the gambling problem	418	72.7%	
Familial history of gambling problems	605	25.5%	
**PSYCHIATRIC AND SOMATIC COMORBIDITIES**			
Mood disorders^4^	628	47.6%	
Anxiety disorders^5^	628	37.9%	
Addictive disorders^6^	628	35.2%	
Psychotic syndrome	627	8.0%	
Actual suicidal risk	628	23.7%	
Parkinson’s disease	627	1.1%	
**ADHD antecedents**			
ADHD screening in childhood (WURS-C)	599	20.7%	
ADHD screening in adulthood (ASRS)	599	18.7%	
**PERSONALITY PROFILE**			
**TCI scores**	594		
*Temperament*			
Novelty Seeking			52.6 (18.0)
Harm Avoidance			43.9 (23.4)
Reward Dependence			60.2 (17.7)
Persistence			55.1 (28.5)
*Character*			
Self-Directedness			67.7 (20.0)
Cooperation			73.9 (15.2)
Self-Transcendence			31.4 (22.4)
Antisocial personality disorder	628	3.4%	

^*Notes:1*^ *Loto® and Kéno® are two national lotteries in France.*

^*2*^ *Euromillions is the European lottery.*

^*3*^ *Rapido® is a French game available in bars. The goal is to find 8 out of 20 numbers in a first grid (grid A) and simultaneously a number of four (grid B). The draw frequency of Rapido® is very high, with one draw every two and a half minutes.*

^*4*^ *Mood disorders included: depressive disorders (major depressive episodes or dysthymia) and manic or hypomanic episodes.*

^*5*^ *Anxiety disorders included: panic disorder, agoraphobia, social phobia, obsessive-compulsive disorder, post-traumatic stress disorder and generalized anxiety disorder.*

^*6*^ *Addictive disorders included: substance use disorders, alcohol-use disorders and eating disorders.*

In order to evaluate the evolution of the status of the gamblers, we will analyze data using a multistate Markov model (with 2 possible states -problem or non-problem gamblers- or 3 possible states –NPG, PGWT or PGST). Variables explaining changes in the status of the gamblers will be studied.

In order to identify longitudinal predictors of the key state changes, we will use mixed linear models allowing taking into account the correlation between the different measures of the patients among times of measurement. Factors predicting variation of time until the key state change will be identified using these models.

## Discussion

The mixing of non-problem gamblers and problem gamblers who have not yet sought treatment is one of the main strengths of our cohort. Problem gamblers who have not yet sought treatment constitute a very rare population in PG research, although they form the key transit state between non-problem gambling and problem gambling with care. Moreover, as noted previously, samples of gamblers assessed over a five-year follow-up are very rare in the literature. The few existing cohort studies have several limitations and fail to identify protective and risk factors which would determine changes of state in the gambling trajectory. It is essential to understand such changes in order to provide appropriate prevention or care programs, and improve our understanding of PG etiology. Our cohort is the first one which is designed to observe the state changes in the gambling trajectory and link them with psychosocial correlates monitored over time, in order to identify the predictive factors of these changes in a prospective and longitudinal manner. Another strength and originality of the project lies in its recruitment of gamblers outside specialized care centers and not only through media or advertising. We also included recruitment directly from the usual gambling places, for about a third of our sample (31%). This method gave us access to a broad spectrum of gambling activities and varying initial levels of gambling practice. Finally, the overall sample size (628) has rarely been achieved for studies with semi-structured interviews (most of the time, studies with such high numbers of participants are telephone-based surveys). Our study design is all the more relevant in that the assessment combines a structured face-to-face interview with self-report questionnaires. Moreover, the monitoring of loss of follow-up over time indicates that the current follow-up participation is just under 60% for the first and second year assessments, and seemed to increase after that (see Additional file 1 for more details). Refusals to continue were the main reason for dropouts during the first-year assessment, and unreachability for the second-year assessment. This result was expected because unreachability was defined as having missed two consecutive follow-up. We expect that the follow-up participation of the third-year and following assessments will increase, because the least motivated participants have withdrawn during the previous steps. However, a participation rate of this order is already a high one for a cohort of this type [34,35].

The study also has several limitations though, especially the restricted amount of data collected. Indeed, some data, which may have had an influence on state changes, were not collected (for example, impulsivity, gambling motivation, etc.). However, we chose to restrict the assessment procedure to a limited number of questionnaires, in an attempt to maximize the acceptation of the procedure by the gamblers. Since recruitment methods were diversified (via the press and in gambling venues), it is also conceivable that the participation of follow-up will be different between gamblers recruited by these different methods. In addition, it could be envisaged that gamblers pursuing or not the follow-up are different populations and that it could possibly bias the results from follow-up data. To verify these possible limitations, we conducted a preliminary analysis to compare participants who withdrew and those who were still in the follow-up on April 30, 2014, that is exactly five years after the first inclusion in the study (detailed results are given in Additional file 2). Participants who withdrew were younger, had a shorter experience of gambling, and gambled more on the Internet. We presume that the younger age explains the shorter experience of gambling (the difference in age is equivalent to the difference in gambling experience) and preference for Internet. It is well-known that the younger the participants, the less persistent they are and the more likely they are to have experienced changes in their living conditions (entry into working life, marriage, relocation, etc.). It is thus not surprising that the younger ones are those who most often withdraw from the study. The other main difference was the mode of recruitment. Participants who are still in the follow-up were much more likely to have been recruited through the press. We expected this result because participants who were recruited via the press had taken the step of contacting us, while for others we took the initiative in approaching them in their gambling places to ask them to participate. No other difference in sociodemographics, gambling habits or psychiatric comorbidities was found between participants who withdrew and those who were still in the follow-up, indicating that the cohort is consistent over time.

Finally, the case–control design of our cohort implies that our sample is not representative of the general population of gamblers (in terms of prevalence of problem gambling in particular). However, the aim was to observe changes of state in gambling practice, and not to establish the prevalence of gambling problems. It was therefore very important to have enough participants in the initial groups to observe these rare changes and thus overcome the limitations mentioned in previous studies [13]. In order to estimate if our sample was closed to the general population, especially in terms of socio-demographic data, we compared the socio-economics of our sample (n = 628) with those of the French national prevalence survey (n = 25 034) [36] (see Additional file 3 for more details). Gamblers from the JEU cohort study shared some socioeconomic characteristics with gamblers from the national prevalence survey. The few differences observed were probably due to the fact that problem gamblers were artificially over-represented in the JEU cohort study (59.2% against 0.3% in the national prevalence survey), while it is a case–control study.

## Conclusions

The JEU cohort study is the first study which proposes to identify the predictive factors of key state changes in gambling practice, such as the emergence of a gambling problem, natural recovery from a gambling problem, resolution of a gambling problem with intermediate care intervention, relapses or care recourse, using a prospective and longitudinal approach. This is the first case–control cohort on gambling which mixes non-problem gamblers, problem gamblers without treatment and problem gamblers seeking treatment in almost equal proportions. We believe that this work may help providing a fresh perspective on the etiology of pathological gambling, which may provide support for future research, care and preventive actions in the field of gambling.

## Additional files

Additional file 1:
**Loss of follow-up on April 30, 2014 (five years after first inclusion in the study).**
Additional file 2:
**Comparisons between participants who dropped out and those who were still in follow-up on April 30, 2014 (five years after first inclusion in the study)** ^**a**^ **.**
Additional file 3:
**Socioeconomics of the JEU Cohort (n = 628) compared with gamblers from French national prevalence survey data (n = 25 034) [** 36 **].**

## Abbreviations

PG: Pathological gambling
NPG: Non-problem gamblers
PGWT: Problem gamblers without treatment
PGST: Problem gamblers seeking treatment
ADHD: Attention deficit hyperactivity disorder
DSM: Diagnostic and statistical manual of mental disorders
MINI: Mini International Neuropsychiatric Interview – fifth version
SOGS: South Oaks gambling screen
GABS-23: Gambling attitudes and beliefs survey - revised version
TCI-125: Temperament and character inventory – 125
WURS-C: Wender-Utah rating scale-child
ASRS-1.1: Adult ADHD self-report scale
