# Supplementary material for: Study protocol: the JEU cohort study – transversal multiaxial evaluation and 5-year follow-up of a cohort of French gamblers
Source: BMC Psychiatry. 2014 Aug 20;14:226. doi: 10.1186/s12888-014-0226-7 (PMC4147162; doi:10.1186/s12888-014-0226-7)
Supplement: Additional file 1: — Loss of follow-up on April 30, 2014 (five years after first inclusion in the study). [file 12888_2014_226_MOESM1_ESM.doc]

**Additional file 1**: loss of follow-up on April 30, 2014 (five years after first inclusion in the study)

|  | **Baseline assessment**  (*completed*) | **First year assessment**  (*completed*) | | | | **Second year assessment**  (*completed*) | | | | **Third year assessment**  (*in progress*) | | | | **Fourth year** **assessment**  (*in progress*) | | | | **Fifth year assessment**  (*not yet started*) | | | |
| --- | --- | --- | --- | --- | --- | --- | --- | --- | --- | --- | --- | --- | --- | --- | --- | --- | --- | --- | --- | --- | --- |
|  | number completed | starting size | | number completed | | starting size | | number completed | | starting size | | number completed | | starting size | | number completed | | starting size | | number completed | |
| NPG | 256 | 256 | | 148 | | 229 | | 134 | | 157 (100) | | 71 | | 148 (27) | | 18 | | 144 (0) | | 0 | |
| PGWT | 169 | 169 | | 101 | | 143 | | 87 | | 110 (37) | | 25 | | 109 (12) | | 5 | | 107 (0) | | 0 | |
| PGST | 203 |  | | | | | | | | | | | | | | | | | | | |
| **TOTAL** | 628 | 425 | | 249 | | 372 | | 221 | | 267 (137) | | 96 | | 257 (39) | | 23 | | 251 (0) | | 0 | |
|  | | | | | | | | | | | | | | | | | | | | | |
|  |  | unreachable | refusal to continue | | death | unreachable | refusal to continue | | death | unreachable | refusal to continue | | death | unreachable | refusal to continue | | death | unreachable | refusal to continue | | death |
| number of dropouts in NPG group |  | 0 | 26 | | 1 | 63 | 9 | | 0 | 9 | 0 | | 0 | 4 | 0 | | 0 | 0 | 0 | | 0 |
| number of dropouts in PSWT group |  | 0 | 26 | | 0 | 29 | 3 | | 1 | 1 | 0 | | 0 | 2 | 0 | | 0 | 0 | 0 | | 0 |
|  | | | | | | | | | | | | | | | | | | | | | |
| **% of follow-up participation for the whole sample** |  | 58.6% | | | | 59.4% | | | | 70.1% | | | | 59.0% | | | |  | | | |

*Notes:*

*When the assessment is shown as “completed”, the whole sample was contacted to carry out the related assessment. When the assessment is shown as “in progress”, only some of the participants were contacted to carry out the related assessment (i.e. the anniversary date of their inclusion has been exceeded by more or less 2 months). When the assessment is shown as “not yet started”, no participants have been contacted to carry out the assessment.*

*The starting size is the size of the group minus the previous follow-up dropouts. When the assessment is in progress, the number between brackets is the number of participants for whom follow-up might have been completed by the present date. The number completed is the number of assessments which have been completed.*

*The percentage of follow-up participation reflects the ratio between the number of assessments completed and the starting size. When the assessment is in progress, the percentage of follow-up participation is calculated as the ratio between the number of assessments completed and the number of participants for whom follow-up might have taken place. However, there is a 2 month delay to carry out the follow-up and then the percentage of follow-up participation might have been underestimated for the assessments in progress.*
