# Supplementary material for: Study protocol: the JEU cohort study – transversal multiaxial evaluation and 5-year follow-up of a cohort of French gamblers
Source: BMC Psychiatry. 2014 Aug 20;14:226. doi: 10.1186/s12888-014-0226-7 (PMC4147162; doi:10.1186/s12888-014-0226-7)
Supplement: Additional file 2: — Comparisons between participants who dropped out and those who were still in follow-up on April 30, 2014 (five years after first inclusion in the study) a . [file 12888_2014_226_MOESM2_ESM.doc]

**Additional file 2**: comparisons between participants who dropped out and those who were still in follow-up on April 30, 2014 (five years after first inclusion in the study)^a^

|  | Participants still in the follow-up  M (sd) or%  N = 251 | Participants dropped out  M (sd) or%  N=174 | Test-statistic^b^ | p-value^c^ |
| --- | --- | --- | --- | --- |
| **SOCIODEMOGRAPHICS** |  |  |  |  |
| Gender (males) | 56.6% | 63.8% | 2.23 (χ²) | 0.135 |
| Age (years) | 46.0 (12.9) | 42.4 (13.9) | -2.75 (t) | **0.006**** |
| Monthly income (€) | 1732.8 (2639.7) | 1669.9 (1474.6) | 21134.50 (U) | 0.951 |
| Professional activity (working) | 58.8% | 60.3% | 0.10 (χ²) | 0.750 |
| Marital status (living alone) | 50.6% | 59.0% | 2.89 (χ²) | 0.089 |
|  |  |  |  |  |
| **GAMBLING HABITS** |  |  |  |  |
| Monthly gambling expenditure (€) | 243.0 (381.9) | 425.9 (1257.5) | 20174.50 (U) | 0.240 |
| Duration since the onset of gambling (years) | 25.1 (12.3) | 21.7 (11.9) | -2.81 (t) | **0.005**** |
| Maximum frequency of gambling  *Less than once a month*  *Less than once a week*  *Once a week*  *More than once a week* | 11.2%  21.1%  18.3%  49.4% | 10.9%  25.3%  21.3%  42.5% | 2.28 (χ²) | 0.516 |
| Gambling preferentially on the Internet | 8.4% | 3.1% | 4.99 (χ²) | **0.025*** |
| Number of positive diagnosis criteria for PG | 2.6 (2.6) | 2.4 (2.7) | -0.917 (t) | 0.360 |
| Denial of the gambling problem^d^ | 10.4% | 12.1% | 0.30 (χ²) | 0.582 |
|  |  |  |  |  |
| **PSYCHIATRIC COMORBIDITIES** |  |  |  |  |
| Mood disorders^e^ | 50.6% | 42.5% | 2.69 (χ²) | 0.101 |
| Anxiety disorders^f^ | 40.2% | 36.8% | 0.52 (χ²) | 0.472 |
| Addictive disorders^g^ | 31.9% | 33.3% | 0.10 (χ²) | 0.752 |
| Psychotic syndrome | 5.6% | 8.2% | 1.47 (χ²) | 0.225 |
| Actual suicidal risk | 18.7% | 18.4% | 0.01 (χ²) | 0.931 |
| Antisocial personality disorder | 3.2% | 5.7% | 1.63 (χ²) | 0.202 |
|  |  |  |  |  |
| **METHODOLOGICAL VARIABLES** |  |  |  |  |
| Mode of recruitment (by press) | 66.9% | 30.5% | 55.98 (χ²) | **<0.001***** |

*Notes:*

*Significance tests compare means/frequencies between* *participants who dropped out and those who were still in follow-up on 30 April, 2014. Comparisons were performed using Chi-squared tests for categorical variables and Student or Mann-Whitney tests for continuous variables, depending on the normality of the variable.*

*PG: Pathological Gambling*

*M: Mean; SD: Standard Deviation; %: Frequencies.*

*^a^ The database consisted of 425 gamblers eligible for follow-up (belonging to NPG and PGWT groups at baseline).*

*^b^ χ²-statistic is given when χ² tests were carried out, t-statistic for Student tests and U-statistics for Mann-Whitney tests.*

*^c^ Significant differences (i.e., p value<0.05) are shown in bold. * p value<0.0 ; ** p value<0.01; *** p value<0.001*

*^d^ Denial of the gambling problem is measured by the presence of discordance between self-assessment of a gambling problem (“Have you had a gambling problem in the past 12 months?”) and diagnosis of a gambling problem by the interviewer (number of positive diagnosis criteria for PG greater than or equal to 3).*

*^e^ Mood disorders included: depressive disorders (major depressive episodes or dysthymia) and manic or hypomanic episodes*

*^f^ Anxiety disorders included: panic disorder, agoraphobia, social phobia, obsessive-compulsive disorder, post-traumatic stress disorder and generalized anxiety disorder*

*^g^ Addictive disorders included: substance use disorders, alcohol-use disorders and eating disorders*
