# Supplementary material for: Study protocol: the JEU cohort study – transversal multiaxial evaluation and 5-year follow-up of a cohort of French gamblers
Source: BMC Psychiatry. 2014 Aug 20;14:226. doi: 10.1186/s12888-014-0226-7 (PMC4147162; doi:10.1186/s12888-014-0226-7)
Supplement: Additional file 3: — Socioeconomics of the JEU Cohort (n = 628) compared with gamblers from French national prevalence survey data (n = 25 034) [ 36 ]. [file 12888_2014_226_MOESM3_ESM.doc]

**Additional file 3**: socioeconomics of the JEU Cohort (n = 628) compared with gamblers from French national prevalence survey data (n = 25 034) [36]

|  | JEU Cohort  N = 628 | | | National prevalence survey [36]  N = 25 034 | |
| --- | --- | --- | --- | --- | --- |
|  | Gamblers ^a^  N = 628 | Problem gamblers ^b^  N = 372 | Gamblers ^a^  N = 11 780 | | Excessive gamblers ^c^  N = 79 |
| Gender *(males)* | 66.6% | 74.2% | 52.2% | | 75.5% |
| Marital status *(lives as a couple)* | 50.1% | 52.2% | 67.7% | | 55.2% |
| Educational level (no diploma) | 9.9% | 11.0% | 16.3% | | 36.3% |
| Monthly income (*lower than 1100 €)* | 29.9% | 30.1% | 32.7% | | 57.8% |
|  |  |  |  | |  |
|  | M |  | M | | M |
| Age | 43.4 | 43.2 | 43.3 | | 41.4 |
|  |  |  |  | |  |

*Notes:*

*^a^ Gamblers are those who have declared gambling in the past 12 months; the “gamblers” category includes problem and non-problem gamblers*

*^b^ Problem gamblers are those who displayed 3 or more positive diagnosis criteria in the PG section of DSM-IV; thus, they include at risk & pathological gamblers.*

*^c^ Excessive gamblers are those who displayed a score of 8 or more in the Canadian Problem Gambling Index (CPGI)* [Ferris J, Wynne H: L’indice canadien du jeu excessif : Rapport final. In Centre canadien de lutte contre l’alcoolisme et les toxicomanies. ; 2001:1–72]
